# Time series forecasting of red blood cell demand in hematology patients using SARIMA and exponential smoothing models: a retrospective analysis in a Chinese tertiary hospital

**DOI:** 10.3389/fmed.2025.1582277

**Published:** 2025-11-24

**Authors:** Jusong Liu, Qiong Yuan, Liang Cai, Zhi Cai, Xuemei Xu

**Affiliations:** 1 Department of Transfusion, Zigong First People's Hospital, Zigong, Sichuan, China; 2 Department of Transfusion, The Affiliated Hospital of Southwest Medical University, Luzhou, Sichuan, China

**Keywords:** SARIMA model, ES model, red blood cells usage, model prediction, hematology patients

## Abstract

**Background:**

Red blood cells (RBCs) infusion is very important for the treatment of hematology patients, but how to maintain a balanced state between the supply and demand of RBCs is still a major challenge.

**Objective:**

This study aimed to explore the feasibility of seasonal autoregressive integrated moving average (SARIMA) model and exponential smoothing (ES) model in predicting the clinical demand of RBCs for hematology patients each month.

**Methods:**

Our study collected the monthly RBCs usage data of hematology patients from January 2014 to December 2023 to establish the SARIMA model and ES model, respectively. Then, the optimal model was used to forecast the monthly usage of RBCs from January to June 2024, and we subsequently compared the data with actual values to evaluate the prediction effect of the model.

**Results:**

The best fitting SARIMA model was SARIMA (2,1,0)(1,1,1)_12_, whose *R*^2^ = 0.603, MAE = 37.092, MAPE = 13.693, BIC = 7.896. The best fitting ES model was Winters addition model, whose *R*^2^ = 0.702, MAE = 32.617, MAPE = 12.138, BIC = 7.485. The mean relative errors of two models were 0.085 and 0.159, respectively. The SARIMA (2,1,0)(1,1,1)_12_ model performed better in prediction.

**Conclusion:**

Compared with the ES model, the SARIMA model has a smaller mean relative error in predicting RBCs usage in hematology patients. DM test also verify this result. But in the future, more similar research data are needed to make research more convincing.

## 1 Introduction

The Department of Hematology is a department that diagnoses and treats various blood diseases. Its common disease types are mainly divided into three categories: anemia diseases, tumor diseases and bleeding diseases. Patients with blood diseases often suffer from symptoms such as anemia and bleeding. Therefore, blood transfusion is one of the most commonly used treatments. Timely and effective blood transfusion can not only maintain blood volume, increase hemoglobin concentration, improve the body’s oxygen carrying capacity, and alleviate the condition, but also supplement plasma proteins and other substances, increase the body’s immune ability, and improve the patient’s prognosis. Studies have shown that among all patients who need blood transfusion treatment, more than a quarter are hematology patients, some patients with special blood diseases even need long-term and multiple blood transfusions, this reflects the irreplaceable role of blood transfusion in the treatment of blood diseases (1–3).

RBCs are the most common blood component in transfusion therapy and play a vital role in the treatment of patients with blood diseases (4). However, due to the short storage time, the collection and storage of RBCs by blood stations and blood transfusion departments are greatly limited, resulting in an imbalance between clinical blood demand and the inventory of RBCs in the blood transfusion department. When the blood transfusion department is short of inventory, patients cannot receive blood transfusion treatment in time, which affects their recovery; when the inventory is in excess, the excess RBCs will lead to waste due to exceeding the storage period (5–7). Therefore, if the demand for RBCs in the clinic in a certain period of time in future can be accurately predicted, it will provide a certain basis and reference for blood stations to recruit blood donors and the timing of collecting RBCs. This not only ensures that the demand and supply of RBCs are in balance, but also enables blood stations and blood transfusion departments to allocate and manage RBCs more scientifically, ensure the rational use of RBCs, reduce the waste of RBCs, and allow clinical patients to receive blood transfusions in a timely manner, especially fresh blood component treatment, thereby further promoting improvement of the disease (2, 8–10).

In a broad sense, a time series is a sequence of values formed at various time points, and is one of the indispensable features of many data (11). It exists in many fields, such as economics, environment, meteorology, humanities, and medicine (12–15). The time series model is a tool used to model regression problems. The entire modeling process has only one feature, time, and the prediction process of the time series model is to use the feature of time to predict the continuous value of the corresponding time series object. It mainly studies the changing law of the time series itself. Time series can be divided into different categories according to the number of research objects, continuity, statistical characteristics, and distribution patterns. There are different analysis methods for different time series (16–18).

SARIMA model is a time series analysis method that can scientifically extract and analyze the seasonal effect, trend effect and the mutual influence of some confounding factors of the time series. It was first proposed by Box and Jenkins in 1970 and was originally designed for economics. ES model is another method which predicts the future by taking a weighted average of past data in a time series. It is a further development and improvement of the weighted moving average. ES model can integrate the information of time series and perform non-equal weight processing on data of different time periods, it is also easy to operate and adaptable. Based on the above advantages, the ARIMA model and ES model is widely used in the prediction of data such as morbidity, mortality, number of hospitalizations and drug demand in medical fields (19–22).

In the past research, Guo (23) used the ARIMA model to predict RBCs demand in pediatric patients. In order to reduce the stability of the platelet supply chain, Bahareh (24) used the ARIMA model to predict the platelet dosage. Volken (25) used the ES model to estimate the trends of whole blood donation and RBCs infusion in a certain area, Han (26) used the ES model to effectively predict the blood components supply in Taiwan’s blood center. While their study applied the models to prediction of blood component dosage and showed good results, none have specifically modeled RBCs demand among hematology patients. Therefore, this study takes the RBCs usage data of the hematology patients as a time series, uses the SARIMA model and ES model to predict their RBCs demand, this is of great significance for them to be able to receive blood transfusion treatment stably.

## 2 Data and methods

**Figure 1:**
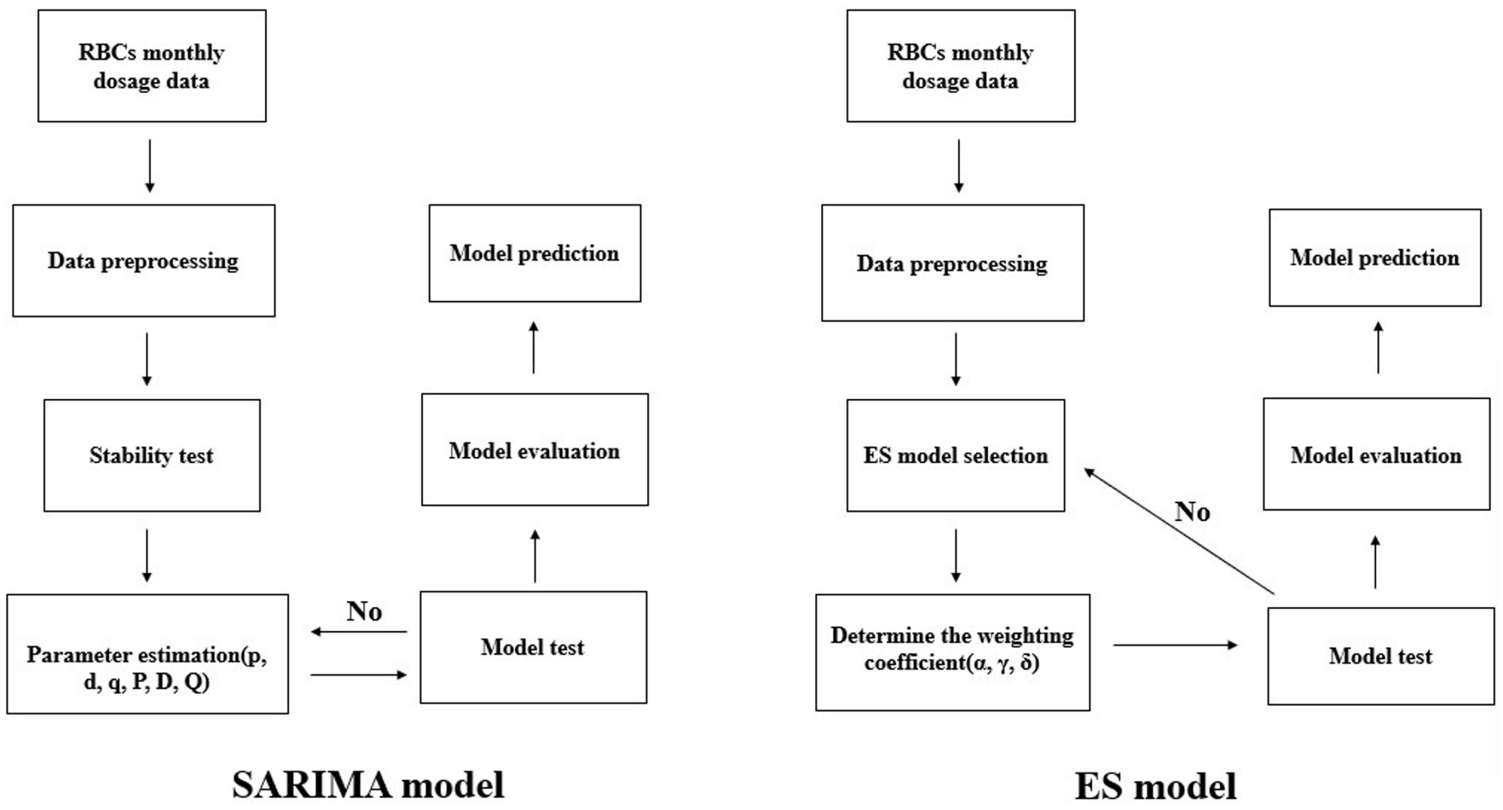
Model construction flow chart.

The construction process of the SARIMA model and ES model is shown in Figure 1. The specific process from data statistics and processing to model prediction will be explained in the following content.

### 2.1 Study sample

The samples included in this study were derived from the information management system of the blood transfusion department of a large general hospital in Sichuan Province. These retrospective transfusion data included the total number of RBCs used by hematology patients each month from January 2014 to June 2024. The types of RBCs included suspended RBCs, washed RBCs, leukocyte-depleted suspended RBCs, irradiated leukocyte-depleted suspended RBCs, and frozen–thawed glycerol-depleted RBCs. The blood types included type A, type B, type O, and type AB. The specific operation process is to use the data from January 2014 to December 2023 as the original sequence for analysis, the data from January 2024 to June 2024 is used to verify the fitting effect of the model. The amount of RBCs is expressed in U. One unit of RBCs (U) is obtained from 200 mL of whole blood. In addition, the specifications of RBCs are mainly divided into three specifications: 1 u, 1.5 u, and 2 u, some newborns may use 0.25 u, therefore, monthly counts are reported as decimals.

### 2.2 Model construction

#### 2.2.1 SARIMA model

The core idea of SARIMA model is to use the historical time data of the variable to predict itself by describing the relationship between the current value and the historical value. The model must meet the requirements of stationarity. For a non-stationary sequence, the trend component can be eliminated through a certain number of differential operations to make it a stationary sequence, and then model it using autoregression (AR) and moving average (MA) methods.

The parameters of the SARIMA model mainly include the autoregressive order (p), difference order (d), moving average order (q), seasonal autoregressive order (P), seasonal difference order (D), seasonal moving average order (Q), and seasonal cycle length (s), expressed as SARIMA (p, d, q)(P, D, Q)_s_. For time series analysis, it is very critical to reasonably select the parameters of the SARIMA model, which directly affects the prediction accuracy of the model. Autocorrelation coefficient (ACF) and partial autocorrelation coefficient (PACF) graphs are important tools for selecting SARIMA model parameters. The values of p, q, P, Q are determined by the order corresponding to the tailing or truncation in the ACF and PACF graph, the values of d and D are determined by the number of differences and seasonal differences.

#### 2.2.2 ES model

ES model uses the weighted average of the past values of the series to predict the future value. The characteristic of this method is to give greater weight to recent data, while give smaller weights to distant data. Therefore, the predicted value can not only reflect the latest information, but also reflect historical data information, making the prediction results more realistic.

There are several different forms of ES model, including Simple seasonal ES, Winters additive ES and Winters multiplicative ES. It is controlled by three parameters: smoothing coefficient *α*, trend coefficient *γ* and seasonal coefficient *δ*, and their values range from 0 to 1, the closer the value is to 1, the greater the weight it has in prediction. Choosing the appropriate ES type according to whether the sequence is trendy and seasonal, and then perform the model test, compare each evaluation indicator to find the optimal model.

### 2.3 Model test

Model test mainly includes two parts: model and parameter significance testing. The model and parameters were tested using the *t* statistic. If the calculated *p* < 0.05, the model was considered to have passed the significance test of the model and parameters, which meant that the model structure had reached the simplest level and the established model was valid. The Ljung-Box *Q* test is used to determine whether the residual sequence of the model is a white noise sequence. If the calculated *p* > 0.05, it can be considered that the residual sequence is a white noise sequence, indicating the model fully extracts the information of the time series. Only models that pass the hypothesis test can be used for time series prediction. If multiple models pass the hypothesis test, the model with a larger *R*^2^, smaller mean absolute error (MAE), and smaller mean absolute percentage error (MAPE) is selected as the optimal model. In addition, the Bayesian information criterion (BIC) can be used to assist in model selection. The smaller the BIC value, the better the model fitting effect. The relevant formula is as follows.


$$R^{2}=1-\frac{\sum 2}{\sum 2},$$

where *yi* represents the actual value, $\hat{y}$ i represents the predicted value, $\bar{y}$ represents the mean value, the numerator represents the sum of squared errors between the predicted value and the actual value, and the denominator represents the sum of squared errors between the mean value and the actual value. The larger the *R*^2^, the better the model fit. When the prediction model does not make any mistakes, *R*^2^ is equal to 1.


$$\mathrm{MAE}=\frac{1}{m}\sum_{i=1}^{m}|\hat{y}i-\mathrm{yi}|,\text{MAPE}=\frac{100\%}{m}\sum_{i=1}^{m}|\frac{\hat{y}i-\mathrm{yi}}{\mathrm{yi}}|,$$

where m represents the number of samples. MAE is the average of the absolute errors between the predicted value and the true value, while MAPE is the relative size of the error between the predicted value and the true value. Both MAE and MAPE range from 0 to 1. The smaller their values, the better the model fitting effect.


BIC=kln(n)−2ln(L),

Where *k* represents the number of model parameters, *n* represents the number of samples, and *L* represents the likelihood function.

### 2.4 Model prediction

Both the SARIMA model and the ES model use the mean relative error (MRE) to evaluate the prediction effect of the model.


$$\mathrm{MRE}=\frac{\sum |\hat{y}i-\mathrm{yi}|}{\sum \mathrm{yi}}$$

The smaller MRE, the better the prediction effect of the model.

### 2.5 Statistical analysis

In this study, *α* = 0.05 is used as the test level, and IBM SPSS20.0 version software (IBM Corp, Armonk, NY, USA) is used to analyze the data.

## 3 Results

### 3.1 Cardinality features

**Figure 2:**
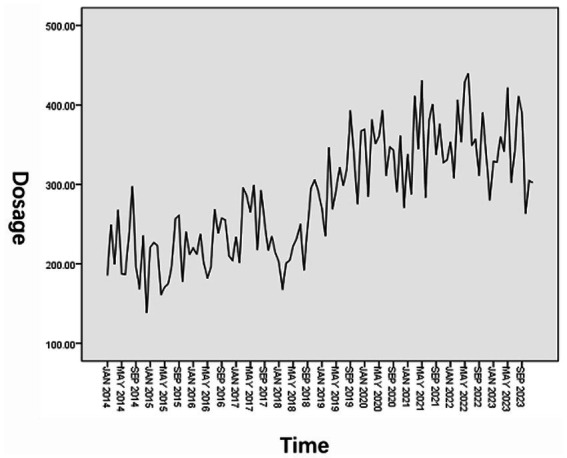
Original sequence diagram of RBCs dosage.

The monthly RBCs usage data of hematology patients from January 2014 to December 2023 were sorted and the original sequence diagram was drawn. The results are shown in Figure 2. It can be seen from the figure that the overall usage of RBCs shows a gradual upward trend. In addition, it is found that the usage is higher in July, August, and September each year, and lower in January, February, and December. The sequence shows obvious periodic and seasonal changes.

### 3.2 Time series analysis and processing

**Figure 3:**
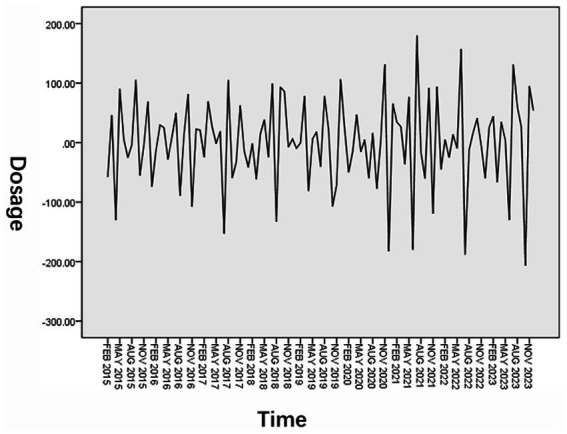
Series diagram after first-order difference and first-order seasonal difference processing.

In order to eliminate the influence of trend and seasonality of the original sequence, it was processed with first-order difference and first-order seasonal difference. The results are shown in Figure 3. By comparison, it is found that after the time series is processed by the first-order difference and the first-order seasonal difference, the upward trend is no longer obvious and to be more stable, each value in the series basically fluctuates around the zero value. Therefore, the seasonal model is used in the subsequent modeling process for research.

### 3.3 Model parameter estimation and model testing

#### 3.3.1 SARIMA model

**Figure 4:**
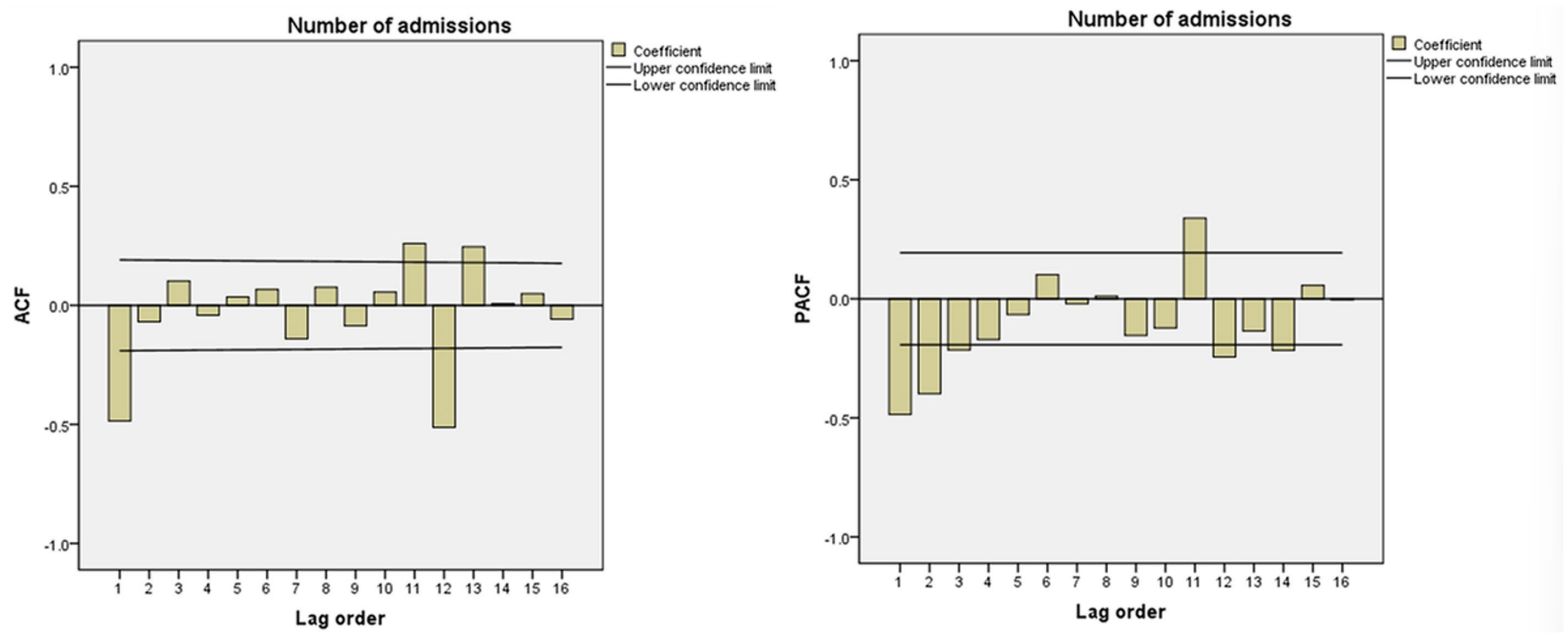
ACF and PACF after first-order difference and first-order seasonal difference.

Observe the ACF and PACF graph after the first-order difference and first-order seasonal difference of the time series. The results were shown in Figure 4. The series was subjected to first-order difference and first-order seasonal difference, d = 1, D = 1, ACF was truncated at 1st lag, q = 1, PACF was tailed at 3rd lag, p = 3. Both ACF and PACF are significantly non-zero at lag 12, P and Q = 1, so the alternative fitting model is tentatively determined to be SARIMA (3,1,1)(1,1,1)_12_.

The t-test was performed on each variable of the SARIMA (3,1,1)(1,1,1)_12_ model parameters, and the results are shown in Table 1. It shows that the AR hypothesis test result of the model failed (*p* > 0.05), so the model needs to be reselected. In order to consider the model more comprehensively, we compared various models by trying from low to high order, then combined various evaluation indicators and hypothesis test result. Finally, we found that only the parameters of the model SARIMA (2,1,0)(1,1,1)_12_ passed the hypothesis test (Table 1), so the final model selected was SARIMA (2,1,0)(1,1,1)_12_, its *R*^2^ = 0.603, MAE = 37.092, MAPE = 13.693, BIC = 7.896 (Table 2).

**Table 1:** Parameter estimation and hypothesis testing results of SARIMA alternative models.

Model		Estimate	SE	*t*	*p*
SARIMA (3,1,1)(1,1,1)_12_	AR	0.173	0.154	1.123	0.264
	MA	0.803	0.180	4.471	<0.001
	SAR	−0.324	0.156	−2.073	0.041
	SMA	0.478	0.154	3.096	0.003
SARIMA (3,1,0)(1,1,1)_12_	AR	−0.137	0.103	−1.323	0.189
	MA	–	–	–	–
	SAR	−0.331	0.156	−2.123	0.036
	SMA	0.460	0.153	2.995	0.003
SARIMA (2,1,1)(1,1,1)_12_	AR	−0.156	0.141	−1.102	0.273
	MA	0.587	0.157	3.744	<0.001
	SAR	−0.293	0.158	−1.855	0.067
	SMA	0.496	0.155	3.208	0.002
SARIMA (2,1,0)(1,1,1)_12_	AR	−0.417	0.092	−4.544	<0.001
	MA	–	–	–	–
	SAR	−0.371	0.151	−2.461	0.016
	SMA	0.445	0.151	2.953	0.004

AR, autoregressive; MA, moving average; SAR, seasonal autoregressive; SMA, seasonal moving average; SE, standard error.

**Table 2:** Evaluation indicators of selected model.

Model	*R* ^2^	MAE	MAPE	BIC	Q statistic	*p*
SARIMA (2,1,0)(1,1,1)_12_	0.603	37.092	13.693	7.896	12.684	0.552

**Figure 5:**
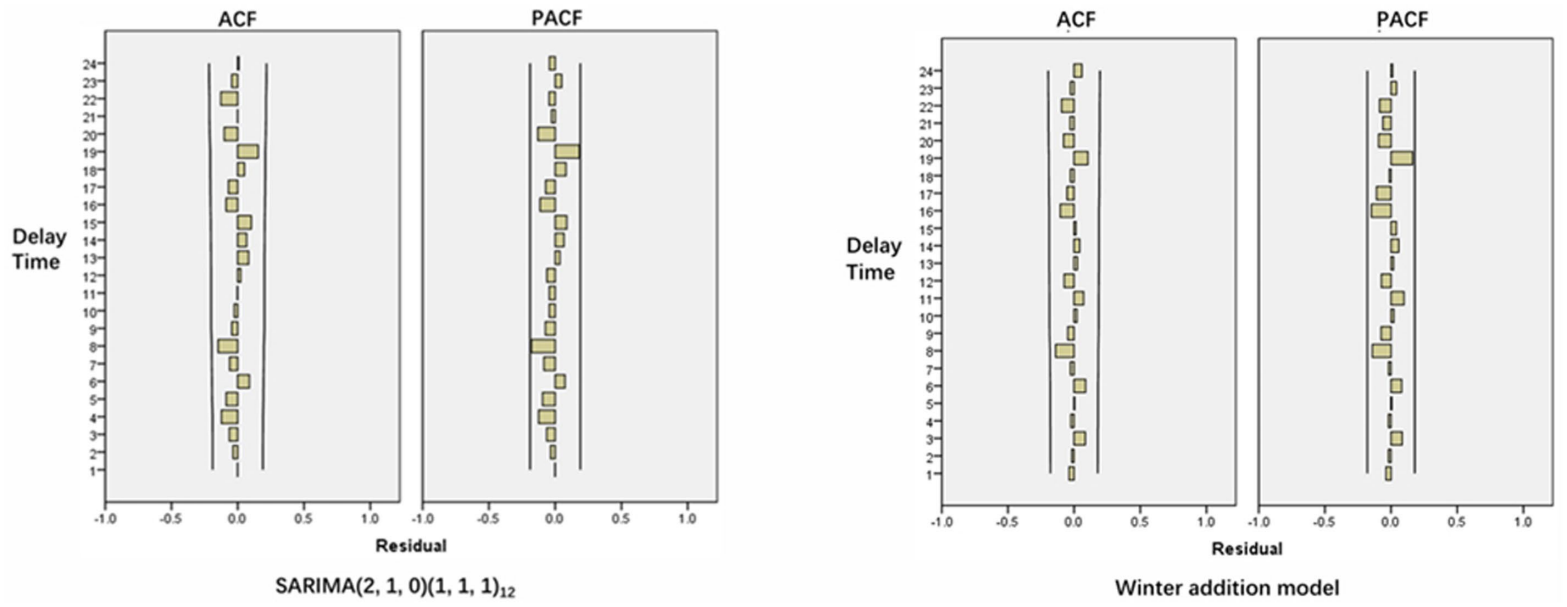
ACF and PACF of model residual sequence.

**Figure 6:**
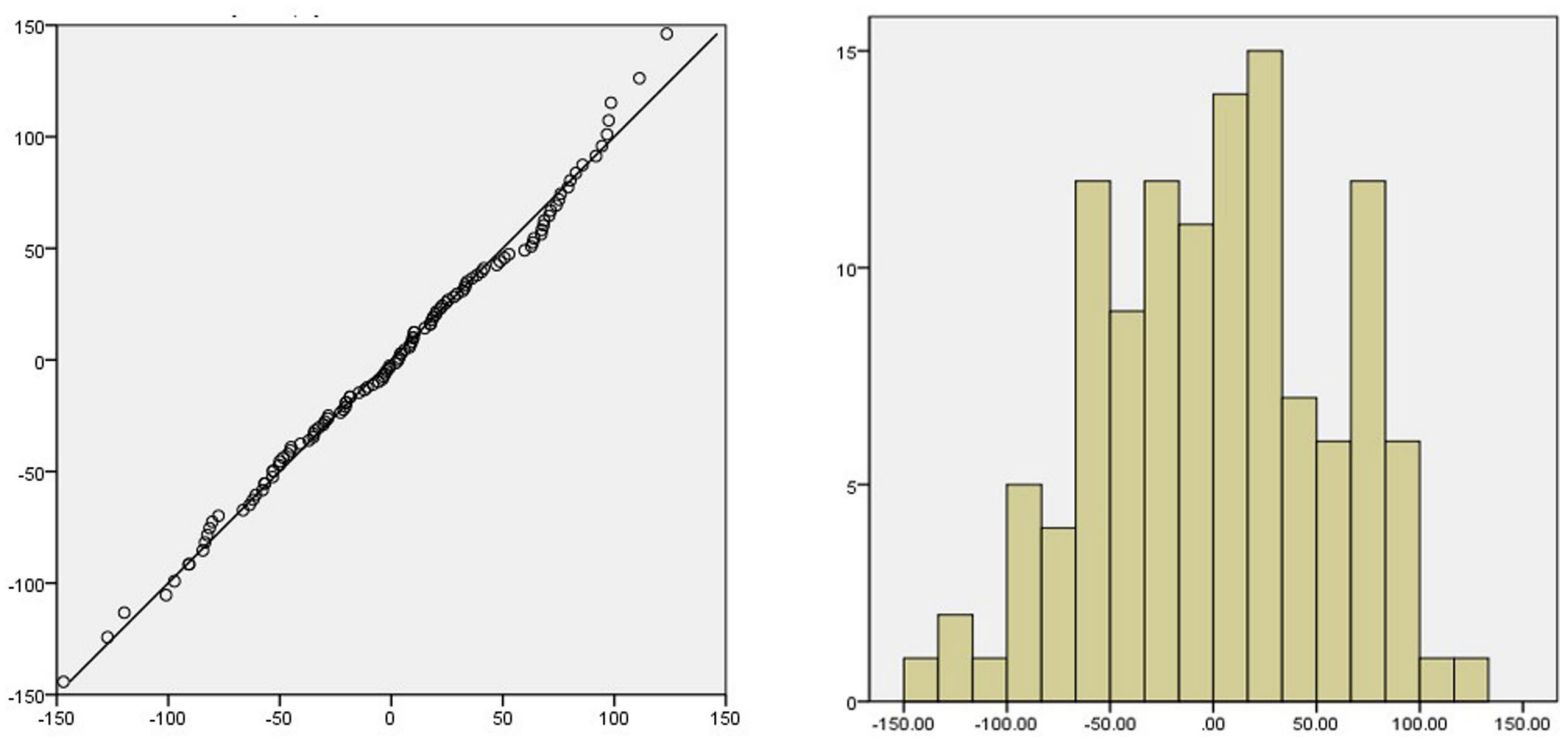
Q-Q plot and histogram of residual sequence.

From the ACF and PACF diagrams of the SARIMA (2,1,0)(1,1,1)_12_ residual sequence (Figure 5), it can be seen that the values of the residual sequence of the model basically fall within the 95% confidence interval (CI). Q-Q plot and histogram show that the residual sequence is basically located on a straight line (Figure 6) and Ljung-Box *Q* test results show that the *Q* statistic is 12.684, *p* > 0.05, indicating that the model residual sequence does not have autocorrelation and partial autocorrelation, meets the randomness assumption, and is a white noise sequence. Therefore, it is believed that the establishment of this model is appropriate and can be used to predict the amount of RBCs.

#### 3.3.2 ES model

Different seasonal ES models are established respectively, and the hypothesis test results of each model are shown in Table 3. There were statistical differences in Alpha among the three models (*p* < 0.05). The simple seasonal model had no statistical significance in Delta (*p* > 0.05). The Winters additive model had no statistical significance in Gamma and Delta (*p* > 0.05). The Winters multiplicative model had no statistical significance in Gamma (*p* > 0.05), but had statistical differences in Delta (*p* < 0.05). *p*-value close to 1 indicates that the model has little statistical difference at this parameter level.

**Table 3:** Statistical analysis of different ES models.

Model	Indicator	Estimate	SE	*t* value	*p*
Simple seasonal model	Alpha	0.200	0.050	3.966	<0.001
	Delta	0.001	0.065	0.001	0.999
Winters additive model	Alpha	0.224	0.060	3.720	<0.001
	Gamma	0.001	0.009	0.137	0.892
	Delta	0.001	0.056	0.018	0.986
Winters multiplicative model	Alpha	0.176	0.049	3.578	<0.001
	Gamma	0.005	0.018	0.284	0.777
	Delta	0.203	0.070	2.905	0.004

SE, standard error.

By comparing the *R*^2^, MAE, MAPE and BIC values of the three models, it can be found that the Winters additive model is the optimal model (Table 4), the *R*^2^ of the model fitting parameters is the largest, the MAE and MAPE are the smallest. The Ljung-Box *Q* test shows that the statistic is 9.047, *p* > 0.05, indicating that the residual sequence after data fitting does not have autocorrelation and partial autocorrelation, it is a white noise sequence. From the ACF and PACF diagrams of the residual sequence, it can be seen that each value of the residual sequence falls within the CI (Figure 5), so the model can be used to predict the amount of RBCs.

**Table 4:** Evaluation indicators of different ES models.

Model	*R* ^2^	MAE	MAPE	BIC	*Q* statistic	*p*
Simple seasonal model	0.696	32.907	12.190	7.455	8.842	0.920
Winters additive model	0.702	32.617	12.138	7.485	9.047	0.875
Winters multiplicative model	0.652	34.927	12.868	7.639	9.063	0.874

### 3.4 Model prediction

**Figure 7:**
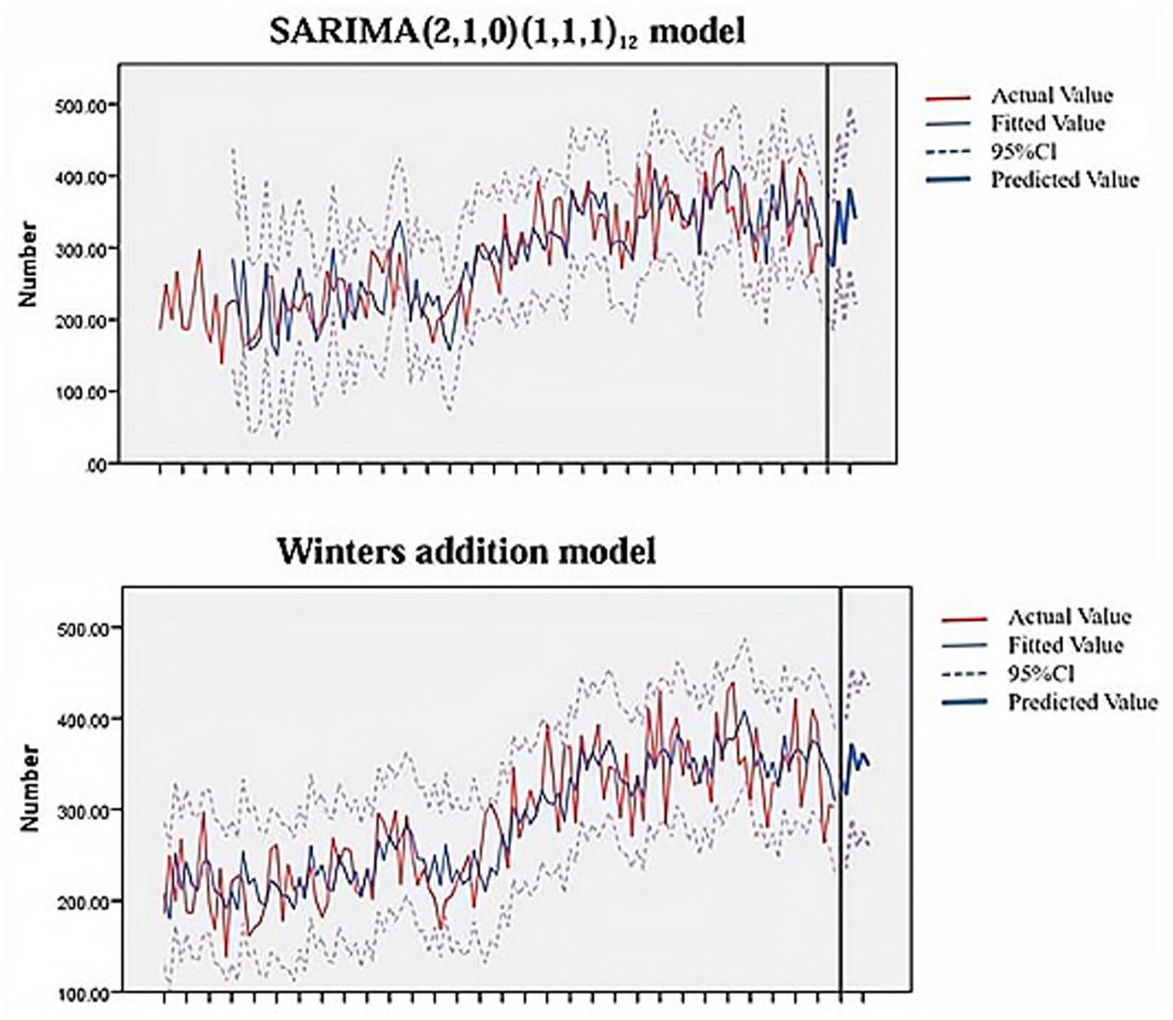
Model prediction fit plot.

The SARIMA (2,1,0)(1,1,1)_12_ model and the Winters addition model were used to predict the RBCs dosage from January 2024 to June 2024, respectively. The results are shown in Figure 7 and Tables 5, 6. As can be seen from Figure 7, the curve change trend of predicted values and actual values in the fitted graphs of the two models is basically consistent and the empirical coverage is 100%. The MRE of the SARIMA (2,1,0)(1,1,1)_12_ model and the Winters addition model are 0.085 and 0.159, respectively (Table 5). By comparing the size of MRE, the SARIMA (2,1,0)(1,1,1)_12_ model has better prediction effect. The Diebold-Mariano (DM) result shows that the *p* < 0.05, indicating that there is a significant difference in the accuracy of SARIMA prediction and ES prediction. From the perspective of the sum of square errors, the sum of square errors of SARIMA’s prediction is smaller than ES, indicating that ARIMA performs better on this data.

**Table 5:** Prediction of RBCs usage by SARIMA (2,1,0)(1,1,1)_12_ model and Winters additive model.

Model	Time	Actual value (U)	Predicted value (U)	LCL	UCL	MAE	MAPE	95%CI	MRE
SARIMA (2,1,0)(1,1,1)_12_	January 2024	269.5	291	204	377	25.46	8.46	(11.94, 38.98)	8.5%
	February 2024	264	273	184	362				
	March 2024	299.25	345	271	459				
	April 2024	281	305	197	413				
	May 2024	347.5	382	269	495				
	June 2024	322	340	221	459				
Winters additive model	January 2024	269.5	338	259	416	44.96	15.84	(22.26, 67.66)	15.9%
	February 2024	264	315	235	396				
	March 2024	299.25	348	260	436				
	April 2024	281	343	258	427				
	May 2024	347.5	361	275	447				
	June 2024	322	348	260	436				

LCL, lower control limit; UCL, upper control limit.

One unit of RBCs (U) is obtained from 200 mL of whole blood. In addition, the specifications of RBCs are mainly divided into three specifications: 1 u, 1.5 u, and 2 u, some newborns may use 0.25 u, therefore, monthly counts are reported as decimals.

**Table 6:** The Diebold-Mariano test results.

Statistic	*p*	Horizon	Loss function
3.39	0.019	1	Square error

## 4 Discussion

RBCs are one of the most commonly used blood products in transfusion therapy. The transfusion of RBCs can enhance the body’s oxygen-carrying capacity, it also has better clinical efficacy and a lower risk of viral and bacterial infections (7, 27). However, all blood products, including RBCs, are in short supply across the country and it is even more scarce in first-tier cities due to large population flows and abundant medical resources. In addition, due to the short shelf life of blood products themselves and imperfect storage methods, an imbalance in the supply and demand of blood products often occurs. Patients are often unable to receive blood transfusions in time, which delays the progress of their disease treatment. Therefore, it is of great significance to find a suitable method to scientifically and accurately predict the demand for RBCs and other blood products, it will provide a basis and reference for the blood collection in the future (28, 29).

The SARIMA model and ES model both use time as a single variable and fit the best prediction model through repeated identification and analysis of time series (30, 31). They are two classic statistical time series models (32). In this study, the SARIMA model and ES model were used to statistically analyze and model the monthly RBCs dosage of hematology patients from January 2014 to December 2023. Then used the optimal model SARIMA (2,1,0)(1,1,1)_12_ and Winters additive model to predict the monthly usage of RBCs from January to June 2024. The results showed that the mean relative error of the SARIMA model (8.5%) was smaller than ES model (15.9%). From this we can see that the prediction effect of the SARIMA model is better. According to Sarvestani’s research results (33), SARIMA can predict the demand for RBCs within 1 year. In Guo’s study (23), they used the SARIMA model to predict the RBC dosage of pediatric patients in a single center in China, with an mean relative error of 6.44% (<10%), which is consistent with our research results. This study also conducted DM test to verify the superiority of the SARIMA model, it is not available in previous studies.

It is worth mentioning that in the process of establishing the SARIMA model, the model selected by the ACF and PACF diagram did not pass the significance test of the model and parameters. According to the operation steps of the relevant research, the optimal fitting model was found by continuously trying from low order to high order. In addition, the model with a smaller BIC value cannot represent the optimal fitting model for the relevant time series prediction. Only when the model pass the significance test, can we ensure that the model extracts sufficient information about the time series, the model structure is as simple as possible and the model establishment is meaningful.

This study also has some limits. First of all, although the sample of this study included data from 10 years from 2014 to 2023, it was only a single-center study. In the future, multi-center research will be needed to obtain data with a larger sample size to make the research results more convincing. Secondly, both the SARIMA model and ES model are built based on existing time series, they do not consider the impact of emergencies such as COVID that occurred in 2020, these mutation points may affect the validity of the model. Thus, medical institutions should constantly update or modify the model according to actual conditions to ensure the accuracy of predictions and provide timely reference for clinical blood use plans.

## 5 Conclusion

By comparing the mean relative error and DM test results, it can be seen that the prediction effect of the SARIMA model is better and is suitable for today’s blood bank management. Medical decision makers can use models to predict the short-term future RBCs usage to ensure the balance between supply and demand, especially when blood resources are limited, it will be meaningful. In addition, the continuously revised model is also necessary, which is the key to ensuring prediction accuracy.

## Data Availability

The original contributions presented in the study are included in the article/Supplementary material, further inquiries can be directed to the corresponding author.
